# From Theory to Practice: An International Approach to Establishing Prehabilitation Programmes

**DOI:** 10.1007/s40140-022-00516-2

**Published:** 2022-02-18

**Authors:** June F. Davis, Stefan J. van Rooijen, Chloe Grimmett, Malcom A. West, Anna M. Campbell, Rashami Awasthi, Gerrit D. Slooter, Michael P. Grocott, Franco Carli, Sandy Jack

**Affiliations:** 1 Macmillan Cancer Support, 89, Albert Embankment, London SE1 7UQ Vauxhall, UK; 2 Allied Health Solutions, Hadlow, Kent UK; 3 Care and Public Health Research Institute, Maastricht University, Maastricht, The Netherlands; 4 School of Health Sciences, University of Southampton, Southampton, UK; 5 University Hospitals Southampton NHS Foundation Trust, Southampton, UK; 6 Academic Unit of Cancer Sciences, Faculty of Medicine, University of Southampton, Southampton, UK; 7 Anaesthesia and Critical Care Research Area, NIHR Biomedical Research Centre, University of Southampton, Southampton, UK; 8 Department of Surgery, St. Mark’s Hospital, Middlesex, UK; 9 School of Applied Sciences, Edinburgh Napier University, Edinburgh, UK; 10 Department of Anaesthesia, McGill University, Montreal General Hospital, Montreal, QC Canada; 11 Maxima Medical Center, Veldhoven, The Netherlands; 12 Anaesthesia and Critical Care Research Unit, University Hospital Southampton NHS Foundation Trust, Southampton, UK; 13 Critical Care Research Area, NIHR Respiratory Biomedical Research Unit, University Hospital Southampton NHS Foundation Trust, Southampton, UK; 14 Integrative Physiology and Critical Illness Group, Clinical and Experimental Sciences, Faculty of Medicine, University of Southampton, Southampton, UK; 15 GICU, University Hospital Southampton NHS Foundation Trust, Southampton, UK; 16 NIHR Southampton Biomedical Research Centre, University of Southampton and University Hospital Southampton NHS Foundation Trust, Southampton, UK

**Keywords:** Prehabilitation, Cancer, Surgery, Business case, Multidisciplinary, Multimodal, Team, Implementation

## Abstract

**Purpose:**

This article focuses on the following:

- The importance of prehabilitation in people with cancer and the known and hypothesised benefits.
- Exploration of the principles that can be used when developing services in the absence of a single accepted model of how these services could be established or configured.
- Description of approaches and learning in the development and implementation of prehabilitation across three different countries: Canada, the Netherlands and the United Kingdom, based on the authors’ experiences and perspectives.

**Recent Findings:**

Practical tips and suggestions are shared by the authors to assist others when implementing prehabilitation programmes. These include experience from three different approaches with similar lessons.

Important elements include the following: (i) starting with a small identified clinical group of patients to refine and test the delivery model and demonstrate proof of concept; (ii) systematic data collection with clearly identified target outcomes from the outset; (iii) collaboration with a wide range of stakeholders including those who will be designing, developing, delivering, funding and using the prehabilitation services; (iv) adapting the model to fit local situations; (v) project leaders who can bring together and motivate a team; (vi) recognition and acknowledgement of the value that each member of a diverse multidisciplinary team brings; (vii) involvement of the whole team in prehabilitation prescription including identification of patients’ levels of risk through appropriate assessment and need-based interventions; (viii) persistence and determination in the development of the business case for sustainable funding; (ix) working with patients ambassadors to develop and advocate for the case for support; and (x) working closely with commissioners of healthcare.

**Summary:**

Principles for the implementation of prehabilitation have been set out by sharing the experiences across three countries. These principles should be considered a framework for those wishing to design and develop prehabilitation services in their own areas to maximise success, effectiveness and sustainability.

## Introduction

### Why Is Prehabilitation Important for People with Cancer?

**Figure 1:**
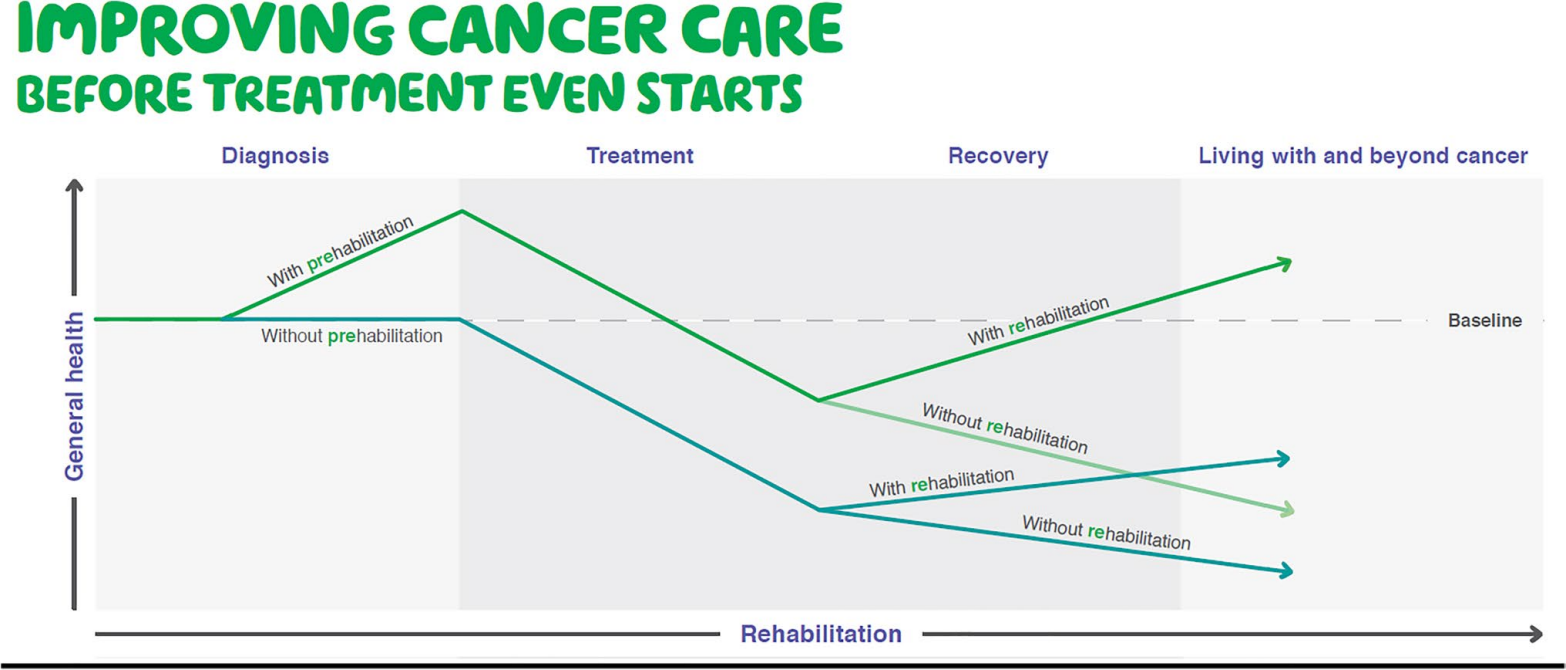
The prehabilitation to rehabilitation continuum. A schematic showing the general health state of patients with cancer over the timeframe from diagnosis of cancer to the post-treatment period. Prehabilitation (before commencement of treatment) and/or rehabilitation (after commencement of treatment) can improve general health at any point of initiation.

Cancer is frequently related to or caused by poor lifestyle factors with a third of cancer cases worldwide being preventable [1]. Prehabilitation focusses on these modifiable risk factors, enabling people to prepare for cancer treatment, with improved outcomes, through promotion of healthy behaviours and need-based prescription of multimodal programmes including exercise, nutrition and psychological interventions. Prehabilitation also includes promoting smoking cessation, encouraging reduced alcohol intake, reviewing polypharmacy and optimisation of chronic conditions such as anaemia and diabetes. Prehabilitation is part of a continuum to rehabilitation [2] (Figure 1) and empowers patients to maximise their resilience to treatment and improve long-term health [3••]. The value of prehabilitation is becoming increasingly recognised within cancer care [4] including improving cardiorespiratory fitness, improving nutritional status, improving aspects of neuro-cognitive function, providing a teachable moment to promote healthy behaviours, enhancing recovery following treatment, reducing post treatment complications and reducing length of stay in hospital [3••].

### When is Prehabilitation Important for People with Cancer?

Prehabilitation should commence at the point of a cancer diagnosis, if not before e.g., at screening. It is useful to consider the current cancer pathway and how the pathway could be redesigned to enable incorporation of prehabilitation [5]. Prehabilitation typically lasts 2–6 weeks depending on the length of time between diagnosis and starting treatment whether surgery and/or radiotherapy and/or chemotherapy and can continue throughout treatment. Prehabilitation interventions can still be effective if begun as little as two weeks prior to treatment [6].

### What Does Prehabilitation Look Like for People with Cancer?

Prehabilitation involves a multimodal approach including:

- structured and personalised aerobic, resistance and flexibility/balance training to minimise/prevent impairments and enhance physical fitness.
- dietary interventions to counteract the potential catabolic state and to support anabolism in synergy with exercise.
- anti-stress interventions to foster resilience and self-efficacy;
- cessation of adverse lifestyle habits (*e.g.*, alcohol, smoking)
- Optimisation of concurrent disease/comorbidities e.g. anaemia, diabetes [7•]

Prehabilitation interventions can be delivered within a framework of Universal, Targeted or Specialist prehabilitation interventions [3••].

### How Should Prehabilitation Be Implemented for People with Cancer?

**Figure 2:**
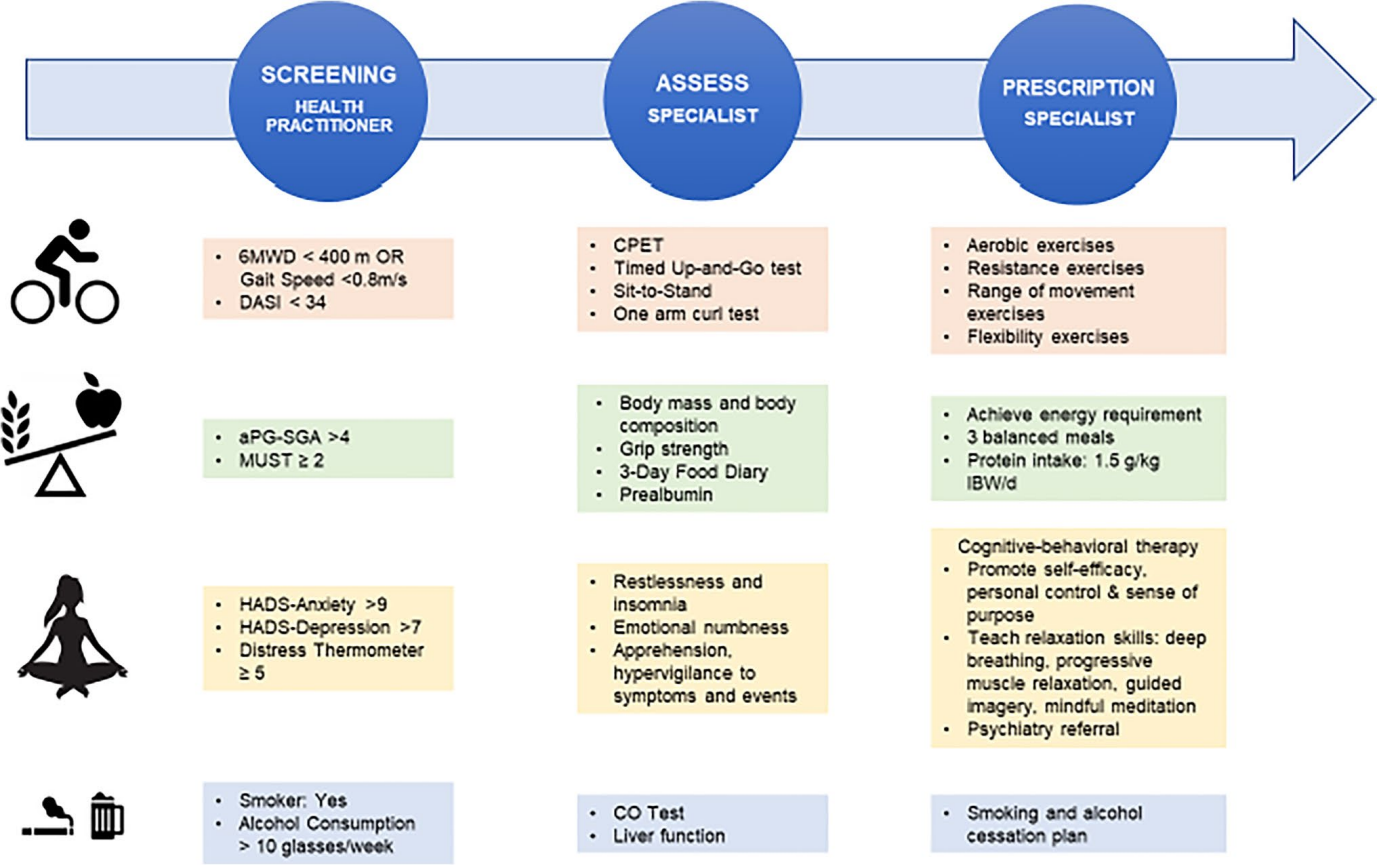
Screening, assessment and prescription of prehabilitation interventions. Patients referred to the prehabilitation programme are screened initially and if necessary, assessed to determine the degree of modifiable impairment(s) before initiation of multimodal intervention. The prescription is individually based. Progression is evaluated regularly. 6MWD, 6-min Walking Distance; aPG-SGA, abridged Patient Generated Subjective Global Assessment; CO, carbon monoxide; CPET, Cardiopulmonary Exercise Testing; DASI, Duke Activity Score Index; HADS, Hospital Anxiety and Depression Scale; IBW, ideal body weight; MUST, Malnutrition Universal Screening Tool.

Patients should be screened and assessed for physical activity and physical fitness, nutritional state, use of alcohol and tobacco, comorbidities and psychological support [3••, 8, 9, 10] in advance of interventions being commenced (Figure 2). Patients may have different degrees of risk for any given component of the intervention and therefore may require different levels of support for each element, within a framework of the Universal, Targeted or Specialist prehabilitation interventions.

When comparing the costs of delivering prehabilitation per patient in each country, the overall range is in the region of £300–400 per patient in the UK, +/− €1000–1200 in the Netherlands and $1500 in Canada. These costs support the screening, assessment and interventions which could be any combination of the following practitioners within the multidisciplinary team: anaesthesiogists, clinical psychologists, dietitians, exercise physiologists, fitness professionals, gastroenterologists, geriatricians, kinesiologists, nurses, occupational therapists, oncologists, physicians, physiotherapists, support workers and surgeons.

### Who Can Benefit from Prehabilitation in People with Cancer?

A comprehensive evidence review [11] identified that benefits from prehabilitation interventions could be seen in people with different tumour types, at different stages of disease, receiving different treatments and across different patient demographics.

There are many similarities in the approaches taken to prehabilitation across different countries. Specific insights and learning from each country are shared.

## Canada

### Idea

The prehabilitation programme was conceptualised at McGill University in 2007. The results of randomised controlled trials supported the contention that the synergistic effect of exercise training, nutrition optimisation and stress management can impact positively on functional capacity before surgery with improvement sustained after surgery. As exercise is a potent anabolic factor, adequate nutrition is necessary to stimulate protein synthesis thus leading to increase in muscle mass and force. Similarly, the adverse impact of anxiety and depression on functional capacity limits patient engagement and resiliency; therefore, stress management and counselling empower patients and encourage participation in their prehabilitation prescription.

#### Suggestion

To commence a prehabilitation programme, centres should review the literature, start with a small group of patients, have a champion surgeon who will support the endeavour and have passion and determination to succeed.

### Team

As the prehabilitation programme evolved, a dietitian and psychosocial trained personnel in stress management were included in the team, along with a perioperative physician and two exercise physiologists. A smoking cessation technician, an internist and a geriatrician were consulted when needed. Graduate (Master, PhD and Postdoctoral) students in exercise science, nutrition, psychiatry and experimental surgery were successful in obtaining grants to undertake research in prehabilitation.

#### Suggestion

Prehabilitation requires a cohesive team where everyone in the team is valued for the contributions to the programme’s success. Each team member assesses the patient, and together, they recommend a prehabilitation prescription for each patient.

### Business Case

Over the last 3 years, we have seen an increase in surgical referrals reaching 150–200 cases per year.

Initially, we did not have the infrastructure to see all these referrals as we had limited staff that comprised of two full-time exercise physiologists, a part-time dietitian, two volunteer retired psychosocial-trained nurses for stress management and one volunteer for social and administration activities (website, newsletter, payroll). These activities were funded following set up of a non-profit foundation for the perioperative programme, with donations from patients and other charitable foundations. Additionally, an annual gala dinner has been able to raise sufficient funds to cover the present costs. Currently, there is considerable interest from the hospital in upgrading the prehabilitation unit to a clinic with full access to the hospital network.

#### Suggestion

Convincing the hospital management that prehabilitation is saving money might be hard work at the beginning; however, they will help you as you show the value, cost-effectiveness and patient-centred nature of the programme. Do not undervalue the help of patients who can act as programme ambassadors.

### Programme

We developed a framework to screen for at-risk patients—including the frail, the sedentary, the malnourished, the socially disadvantaged and those patients scoring high for depression and anxiety, who would have specific impairments requiring detailed selected intervention. A screening procedure has been introduced whereby patients are screened for functional capacity, nutritional status and psychosocial status and prehabilitation prescriptions are tailored.

#### Suggestion

‘One prescription does not fit all’. There is a need to assess patients using validated metrics and plan appropriate individualised interventions to maximise the benefits and to control costs.

### Initial Pilot

Colorectal cancer surgery was chosen as we were familiar with some aspects of the perioperative trajectory. Forty patients were chosen, and results were compared with another group who did not receive prehabilitation.

#### Suggestion

Choose a surgical model you are familiar with and in agreement with a champion surgeon. Prepare a pragmatic, feasible protocol with your team. Identify an outcome that is meaningful to the patient (e.g., functional outcome: walking, able to conduct daily activities; compliance to the protocol, length of hospital stay, return to baseline). Collect and analyse your data carefully (including non-responders) involving the whole team. Evaluate and find solutions to the barriers that patients might encounter when asked to exercise.

### Scaling Up

We identified priorities in our programme and started screening for patients to identify those patients who could be independent, with support from home or in the community gym. Those in need of more personalised and structured prehabilitation are brought to clinic and closely supervised, with progress monitored. A recent tele-prehabilitation project has commenced to reach those patients who are far from the hospital, older, isolated and the socially disadvantaged.

#### Suggestion

Make sure that you know your patients well and feel comfortable they can exercise at home without supervision. Working together with a community centre could be useful when patients cannot or do not wish to travel. Ensure you establish regular contact with the patients in case they need you and to determine whether they are compliant to the exercise training, the nutrition counselling and relaxation techniques.

### Implement Integration with the Preoperative Clinic

Over time our prehabilitation clinic became more integrated with our preoperative clinic and some patients are now directly referred at the time of the preoperative visit. The time interval to surgery might be short. If necessary, the surgeon is informed, and the surgery is put on hold until the patient is considered able to sustain the surgical insult. It is important to develop screening protocols with cut offs (e.g. Dukes Activity Status Index (DASI) less than 34, significant loss of weight, abridged patient generated—subjective global assessment (aPG-SGA) score over 4) whereby patients are referred for evaluation, risk assessment, and if necessary, receive tailored prehabilitation interventions.

## Netherlands

### Idea

A multimodal multiprofessional prehabilitation programme was designed and tested on patients undergoing colorectal cancer surgery at the Maxima Medical Center (MMC) in the Netherlands. The number of postoperative complications was comparable to those across the Netherlands; however, MMC had the vision to enhance its performance. A new prehabilitation programme was designed (Fit4Surgery), consisting of 5 pillars: exercise, nutrition, smoking cessation, mental support, comorbid disease optimisation (e.g., anaemia, diabetes mellitus) and then tested for safety, feasibility and potential effectiveness.

#### Suggestion

Do not start on your own. Seek help, find your believers and champion stakeholders, create a programme that fits your local situation, have all specialties on board (think of the hospital board, management, finance control, health insurance, patient organisations). Start small with a pilot study and scale up.

### Team

Fit4Surgery required the collaboration of 10 medical specialities within the hospital. A surgeon led the multidisciplinary team, which included a dietitian, physiotherapist, gastroenterologist, oncologist, sports physician, psychologist, anaesthesiologist and several nurses. A case manager informed, scheduled and guided the patients throughout their prehabilitation journey. Together with four surgeons, the Dutch Fit4Surgery Foundation was developed to further support (inter) national collaboration. Engagement with the hospital board occurred at an early stage; however, they were not immediately convinced about the potential benefits and were worried by the potentially high development costs. The management team embraced the idea immediately and financially supported a pilot study of 50 patients. A patient foundation also contributed financially to the pilot study. This and two other major patient organisations were asked to further review the content of the programme. The main health insurance company in the region was engaged from the outset.

#### Suggestion

Collaboration with multiple stakeholders is key in prehabilitation. The right project lead is important to keep all believers linked together. Make sure all non-medical and relevant medical team members (i.e., patients/management/clinical staff) are involved.

### Business Case

Fit4Surgery started by setting up an optimal programme together with patients and experts, based on the vision of Value-Based Healthcare (VBHC) [12].

Identifying outcome measures and starting collaboration with management and health insurers enabled potential savings to be calculated e.g., reducing complications by 25% would save €2253 per patient. Costs for the Fit4Surgery programme were calculated at €1010 [13, 14]. These ‘in hospital’ costs and potential savings supported a positive business case, with €1241 cost-saving per patient.

To make Fit4Surgery a sustainable health plan, we also explored the potential additional savings together with the health insurer. These ‘out of hospital’ costs were potential long-term savings for patients and society, including development costs, time investment and non-tangible improvements.

#### Suggestion

Use the Value-Based Healthcare principle as your lever to provide relevant metrics including outcomes and cost savings. Leverage management and others create the real business case.

### Our First Pilot Study

Fifty patients were recruited and included into either the prehabilitation (*n*=20) or control (*n*=30) group, based on availability of the programme at the time of recruitment.

Eighty-six percent of the prehabilitation group recovered to baseline functional capacity within 4 weeks after surgery, compared to 40% of the control group. We also found prehabilitation to be safe for the elderly performing high-intensity training, with a 92% adherence to all training.

#### Suggestion

Decide on the most important outcomes as a multiprofessional team that includes patient representation. Ensure the programme is feasible and fits your local situation and collect pilot data to convince other stakeholders to scale up.

### Implementation and Scaling Up

Data is required to identify further funds, work with insurers and convince the organisational leadership that there is return on investment by funding sufficient staff to keep the prehabilitation team running. The patient journey needs to avoid professionals working in silos. Some elements of the programme could be delivered either in patients’ homes or at local sports facilities.

Our Fit4Surgery foundation has an agreement with the national health authority and representation of all insurers, for reimbursement of the programme for at least 5 years to further investigate the benefits, and to make the Fit4Surgery programme more accessible including in primary care.

#### Suggestion

Create your team of believers and champion stakeholders, ensure you have clinically relevant metrics for the business case, map the patient journey and decide on patient selection, accessibility to the programme and sustainability (i.e., how to proceed after hospital discharge).

## UK

### Stakeholder Engagement

Stakeholder engagement is critical to the design, development, delivery and commissioning of a prehabilitation service.

From our experience essential to success is:

- The patient voice, steer and guidance from the outset and throughout implementation. Patients bring a lived experience, which can help to ensure new programmes are relevant, meaningful and achievable. The Person Based Approach (PBA) is particularly relevant to prehabilitation. The key focus of which is to gain ‘insight into how different people in different situations perceive and execute the behavioural elements of the intervention, why some elements may be particularly necessary or salient to them—or alternatively may be aversive or problematic—and thus how the intervention can be made more attractive, persuasive and feasible to implement’ [15]. Ensuring patient representation from a wide range of socioeconomic and cultural backgrounds is important to ensure acceptability and relevance to all end users.
- Executive/senior sponsorship of the initiative
- Clinical leadership from across professions including oncologists, anaesthesiologists, surgeons, geriatricians, allied health professionals, nurses, psychologists, clinical exercise scientists and clinical researcher/academics
- Involvement of all sectors providing and commissioning health and care services i.e., primary, secondary and tertiary healthcare, third sector including charities and the fitness and leisure industry.

### Patient Complexity

Through triage, multiple factors provide an understanding of the complexity of a patient’s health status, guide clinical decisions on individual recommendations and characterise the risk for exercise related complications. These domains include cardiometabolic status (obesity, diabetes and cardiovascular events such as myocardial infarction or stroke), comorbidities (mental health issues, geriatric issues, autoimmune diseases), treatment toxicities (fatigue, neutropenia, arthralgias, bone metastases) and behavioural characteristics (lack of time, energy, motivation or low self-efficacy). Screening using these domains enables the provider to identify who is able to and will benefit from which components of a prehabilitation service.

### Access to Prehabilitation and Mode of Delivery

Delivery of the majority of prehabilitation programmes is face to face in community and/or hospital settings. Many of these have adapted to virtual/online support during the COVID-19 pandemic. In response to COVID-19, SafeFit [16], a virtual, multimodal, supervised offer, was designed and developed where patients could self-refer through a dedicated Macmillan Cancer Support charity website page.

### Workforce

In the UK, prehabilitation is delivered by different members of a multidisciplinary team. Typically, there are any combination of registered professionals (e.g., dietitians, occupational therapists, physiotherapists, psychologists) and unregistered professionals (e.g., rehabilitation/therapy/cancer support workers, healthcare assistants, fitness instructors).

The SafeFit model [16], a virtual, home-based multimodal service, involves cancer exercise specialists (CESs) who are trained to improving fitness or prevent physical deconditioning. Additional cancer specific training is provided for these cancer exercise specialists in the area of Good Clinical Practice, nutrition, psychological support and behaviour change. Following an initial assessment, the cancer exercise specialists design the exercise prescription and develop a safe, evidence-based, individualised exercise programme aimed at meeting a patient’s needs and preferences. In consultation with the patient, goals for nutrition and psychological optimisation are also set.

### Business Case

Prehabilitation has the potential to offer significant cost-savings, including:

- reducing disutility of care [17], such as complications impacting on length of stay and the associated costs of other clinical conditions.
- reducing the touch points for people with cancer accessing different parts of the healthcare system that they may not need if support is accessed early on.
- enabling earlier return to employment after treatment.

It is important to identify opportunity cost, in that every amount spent on prehabilitation is potentially not spent elsewhere in the patient’s journey or in other parts of healthcare or non-healthcare resources [18•]. Determining the effectiveness of current services is therefore an important starting point with tools available that were developed for services providing rehabilitation and which can equally be used for assessing prehabilitation services [19].

Recent discussions between healthcare service providers and commissioners have identified the following questions:

- How integral is prehabilitation to the whole pathway of care for those with cancer who may have other long-term conditions as well? This is important when commissioning healthcare services at a population level.
- What outcome data is already/could be collected locally? How will benchmarking be enabled between prehabilitation services? How will inequalities be reduced in terms of access to and experience of services, and outcomes achieved?

## Discussion

This paper has shared the experiences across three countries. A summary of a stepwise approach to setting up a prehabilitation service is summarised below.

1. Idea
  Create a multimodal programme together with patients and relevant stakeholders and ensure it fits your local situation.
2. Team
  Create a multiprofessional team with believers, champions, key stakeholders and together design the patient journey.
3. Business case
  Apply Value-Based Healthcare (VBHC) principles, identify where cost-savings are realised, and leverage management for support.
4. Pilot
  Decide on the most important outcomes that need to be measured to inform a business case for sustainable funding. Test your prehabilitation design and numbers (outcomes, cost, cost-savings) to convince others (hospital management, insurers) to scale up.
5. Implement
  Decide on patient selection, accessibility to the programme and strategies to ensure sustainability.

Developing sustainable, clinically effective and cost-effective models of prehabilitation is critical to enable the reach of services to those that require it based on risk stratification and patient complexity. Demonstration of the health economic case is also important with measurement of robust financial and clinical outcomes, including where in the healthcare system the savings will be realised to support sustainability. Longer term, prehabilitation can maintain, or improve, a person’s on-going physical and mental health and well-being by mitigating modifiable risk factors. This may reduce the prevalence of long-term conditions and associated costs including prescribed medications. This necessitates that prehabilitation integrates with primary heathcare to realise the positive impact on population health.

Work is underway in the UK to develop an outline business case and service specification using a value based health care commissioning approach [20]. These principles could be used across countries to support further investment in this vital area of care for people with cancer with the aim of achieving equity for all.
